# A Scoping Review on Goals of Care Discussions in Surgery: How Are We Doing and How Can We Do Better?

**DOI:** 10.1002/wjs.70070

**Published:** 2025-08-23

**Authors:** Amanda Mac, Yerin Lee, Selena Zhang, Abu Sadat Mohammad Nurunnabi, Marina Englesakis, Karen Devon

**Affiliations:** ^1^ Temerty Faculty of Medicine University of Toronto Toronto Ontario Canada; ^2^ Division of General Surgery Department of Surgery University of Toronto Toronto Ontario Canada; ^3^ Dalla Lana School of Public Health University of Toronto Toronto Ontario Canada; ^4^ Library and Information Services University Health Network Toronto Ontario Canada; ^5^ Endocrine Surgery General Surgery Women's College Hospital Toronto Ontario Canada; ^6^ Endocrine Surgery General Surgery University Health Network Toronto Ontario Canada

**Keywords:** goals of care, patient autonomy, patient‐centered care, share decision‐making, surgery, surgical ethics

## Abstract

**Background:**

Discussing GOC is essential to ensuring that patients' treatment recommendations and care plans are aligned with their preferences, priorities, and values. This review aims to characterize the existing literature on the quality, practices, and frameworks of goals of care (GOC) discussions in surgery to identify gaps and propose strategies for improvement.

**Methods:**

MEDLINE, MEDLINE In‐Process/ePubs, Embase, Cochrane Central Register of Controlled Trials, Cochrane Database of Systematic Reviews, Web of Science, Scopus, and ClinicalTrials.Gov were searched using terms related to GOC, surgery, and best practices or education. The search strategy was run from inception to July 29, 2022. Studies regarding the quality of GOC discussions in surgery were included.

**Results:**

The search identified 14,254 articles from which 37 were included for review. Key findings included (1) the reactive nature of GOC discussions and initiating conversations in response to acute health changes, (2) ambiguity around patient autonomy and the surgeon's duty to prioritize surgical treatment, (3) surgeons as curators of information, and (4) tendency of surgeons to provide a set of standard treatment pathways and determine specific care decisions rather than establish understanding of patients' long‐term goals.

**Conclusion:**

Further research is needed to determine best practices for caregiver and next‐of‐kin involvement and expand the diversity of reported experiences to include patients from diverse ethnic backgrounds and genders and individuals from rural and lower‐resource communities. Findings from this review have important implications for improving GOC conversations to ensure they support patient‐centered care.

## Graphical abstract

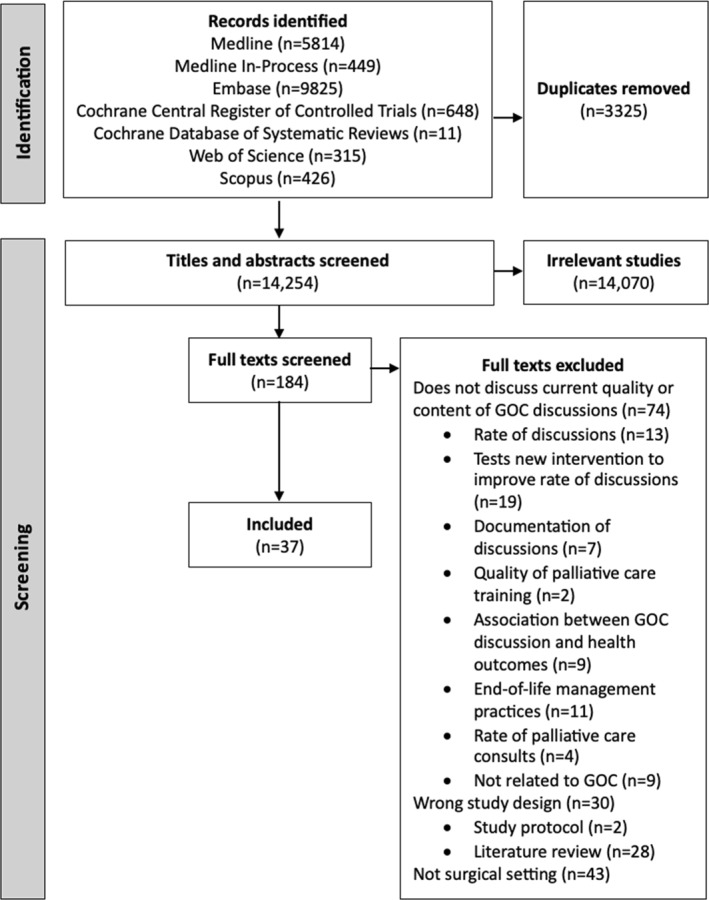

## 1 Introduction

In practice, “goals of care” (GOC) is an umbrella term referring to a range of topics, including clarifying code status, advance care planning, discussing end‐of‐life preferences, or determining a plan for care [1, 2, 3, 4]. In this paper, we define GOC discussions as conversations that include both the sharing of information regarding diagnosis, prognosis, and management and the elicitation and incorporation of patient preferences, priorities, and circumstances. Although the specific content of a GOC discussion may vary, a general approach includes the following key components: assessing the patient's willingness to receive information and their preferred role in decision‐making, understanding the patient's values and future, determining unacceptable health states, exploring preferences for specific life‐sustaining interventions, and updating advance directives [1, 5]. Effective GOC discussions are contingent on clinician comfort and skills; namely, active listening, simplifying complex information, empathy, and offering feasible recommendations that align with the patient's goals and values [5, 6, 7].

Surgical teams are uniquely positioned to engage patients in GOC discussions across various contexts, such as during preoperative planning and consent [8]. Despite this, the quality of GOC discussions in surgical specialties remains inconsistent as does surgeons' understandings of what constitutes true goal‐concordant care [8, 9, 10]. Although the importance of effective GOC discussions is undisputed, surgeons face challenges in conducting comprehensive and meaningful conversations, especially in high‐risk scenarios [8, 11, 12]. Barriers include insufficient time, poor prognostic accuracy, lack of experience, faulty assumptions about the elements of informed consent, and feelings of guilt or responsibility during crises [8, 11, 12, 13, 14]. Absence of GOC discussions can lead to unwanted or nonbeneficial treatments, increased patient and family distress, poor quality of life, and breakdown in trust [8, 9, 10, 11, 12, 13, 14]. With the shift toward personalized medicine and rise in medical advances that prolong life, GOC discussions have become increasingly important in surgical settings [8]. This aim of this scoping review is to summarize the literature on the quality, practices, and frameworks of GOC discussions in surgery to identify gaps and propose strategies for improvement.

## 2 Methods

### 2.1 Study Design

This scoping review was reported in accordance with the Preferred Reporting Items for Systematic Reviews and Meta‐analyses extension for Scoping Reviews (PRISMA‐ScR) statement [15].

### 2.2 Identifying the Research Question

The purpose of this review is to gather and collate the literature on the current state of GOC discussions in surgery. Gathering data on how GOC discussions are conducted in surgical settings will help identify gaps in our current practice and inform recommendations for Improvement. Our research question is as follows: what does the literature reveal about the quality, practices, and frameworks or guidelines of GOC discussions in surgery?

### 2.3 Identifying Relevant Studies

Seven biomedical literature databases and one trial registry were searched by a health sciences librarian (ME) from inception to July 29, 2022:

- MEDLINE (Ovid platform)
- MEDLINE In‐Process/ePubs (Ovid platform)
- Embase Classic + Embase (Ovid platform)
- Cochrane Central Register of Controlled Trials (Ovid platform)
- Cochrane Database of Systematic Reviews (Ovid platform)
- Web of Science Core Collection (Clarivate Analytics)
- Scopus (Elsevier)
- ClinicalTrials.Gov

Each database was searched from its inception using a comprehensive search strategy that was adapted to each database. Our search strategy comprised of controlled vocabulary terms and text words related to GOC, surgery, and best practice guidelines or education and was limited to English language and human subjects. Conference abstracts and/or nonjournal materials were removed at source where possible (Supporting Information S1: Supplement 1). GOC was conceptualized as encompassing any aspect that contributes or pertains to the overarching aims of care for a patient, informed by the patient's values and priorities, established within the clinical context, and used to guide decisions about care [16, 17]. All references identified from our search were compiled into an Endnote file for processing (*n* = 17,488), followed by Covidence for removal of duplicate references and screening.

### 2.4 Study Selection

After removal of duplicate records, a total of 14,254 records were available for screening. We employed Covidence for two‐stage citation screening (title/abstract and full‐text) with dual independent reviewers at each stage (AM, SZ, YL, AN, and KD) [15]. For each stage of screening, records were included if they met all of the following inclusion criteria:

- Full‐text available in English
- Peer‐reviewed empirical research
- Surgical setting
- Focused on any aspect(s) of GOC discussions

At each stage of screening, records were excluded if they met any one of these exclusion criteria:

- Conference proceedings or abstracts
- Dissertations of theses
- Studies focused on pediatric populations (age 0–18)
- Commentaries or position statements
- Literature reviews

**FIGURE 1:**
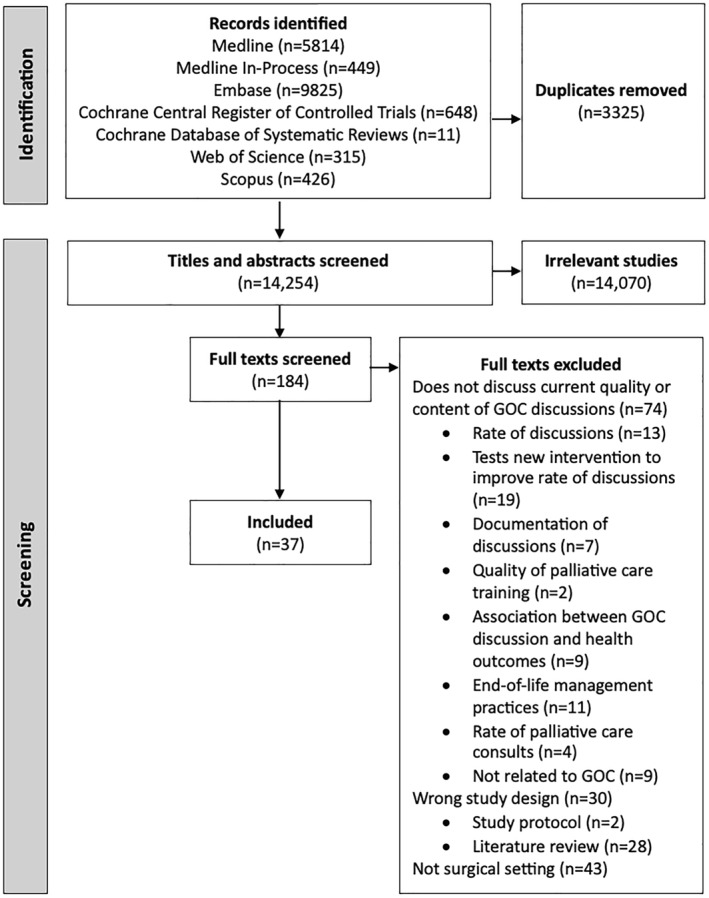
PRISMA diagram outlining screening process, reasons for full text exclusion, and number of records.

The title and abstract screening process resulted in the exclusion of 14,070 records, leaving 184 records for full‐text screening. Of the 184 full texts, 147 were excluded and 37 were included. When conflicts during the screening process occurred, all five reviewers (AM, SZ, YL, AN, and KD) discussed the study in question until reaching consensus. Figure 1 outlines the screening process and reasons for full text exclusion.

### 2.5 Charting the Data

The research team identified key data points to extract from each article: author, year of publication, country of publication, number and type of participants (patients, family, physicians, or trainees), proportion of participants that were surgical (either surgical patients or surgeons/surgical trainees depending on the participant type), demographic information of surgical participants age, sex/gender ratio, race/ethnicity, surgical setting [emergency benign, emergency oncology, elective benign, or elective benign], type of surgery, and academic vs. community setting. Other extraction points involved the purpose and design of the study, whether it was single‐ or multicenter, and the study's main findings. During extraction, each study was read in its entirety and relevant information was charted (Supporting Information S1: Supplement 2) (AM). We used a standardized screening form to apply data extraction consistently (Supporting Information S1: Supplement 2) and all data were reviewed by the senior author (KD).

### 2.6 Collating, Summarizing, and Reporting Results

We conducted a quantitative analysis of the included studies using descriptive statistics, with publication date, geographic location, participant population, surgery setting, methods used, and specific aspects of GOC discussions addressed. Following this, the team engaged in detailed discussion regarding all included articles and Supporting Information S1: Supplement 2 to determine major themes.

## 3 Results

Supporting Information S1: Supplement 2 summarizes the 37 included articles. All studies were published in 2006 or later. The majority (81%) of studies originated from North America, with publications from the USA (*n* = 30) and smaller contributions from Canada (*n* = 4), the United Kingdom (*n* = 3), Denmark (*n* = 1), Switzerland (*n* = 1), and Japan (*n* = 1).

Study populations included surgeons and/or surgical trainees only (*n* = 26), surgical patients and/or family members only (*n* = 8), or both (*n* = 4). Most studies elicited surgeon perspectives on GOC discussions or education in their respective specialties. The age range of participants was between 19 and 102 years old, with four articles focusing solely on the elderly (65 and older). Among studies that included surgeons and/or surgical trainees as participants and recorded the female‐to‐male participant ratio (*n* = 15), five articles had more female participants. Of the 30 studies that included surgeons and/or surgical trainees, three recorded data on race and/or ethnicity. Two had predominantly White participants (71.4% and 80%, respectively), one population was predominantly non‐Hispanic (88.5%), and one study recorded the proportion of Black participants which was 2.9% of the study population. In the 12 studies that included surgical patients and/or family members, four recorded data on race and/or ethnicity. One included only Japanese participants, three were predominantly White (91.7%, 83.1%, and 95.5%), and one noted the percentage of Black participants (8.3%).

Regarding surgical setting, most studies examined GOC discussions in the elective/scheduled oncology setting (*n* = 13), followed by emergency surgery (*n* = 6) and a mix of all settings (*n* = 5). A wide range of surgical specialties were included as outlined in Supporting Information S1: Supplement 2. Most studies were conducted in an academic setting (*n* = 21), followed by mix of both academic and community settings (*n* = 6), and one study in a community setting only.

Of the 37 studies, most focused on experiences and perspectives related to advance care planning, emergency care planning or advance directives (*n* = 12) [18, 19, 20, 21, 30, 31, 32, 38, 39, 43, 44, 49], and end‐of‐life discussions (*n* = 8) [22, 23, 24, 25, 33, 40, 41, 45]. Five studies explored the current knowledge base and educational gaps of surgical trainees and surgeons regarding end‐of‐life care, palliative care, and their experiences with GOC discussions [26, 34, 46, 50, 53]. The remaining studies explored physician perspectives on key elements, barriers, and facilitators of effective GOC discussions (*n* = 6) [27, 28, 29, 42, 51, 54], physician preferences for information sharing and considering GOC of patients (*n* = 4) [35, 36, 47, 48], and paradigms that patients use to understand and decide on perioperative management (*n* = 2) [37, 52].

In terms of methods, most studies used qualitative designs including focus groups (*n* = 4) [42, 43, 45, 52] and interviews (*n* = 14) [19, 20, 21, 28, 29, 30, 35, 36, 37, 38, 44, 47, 49, 51]. Studies also employed ethnographic observation (*n* = 4) [18, 35, 45, 54], surveys with open‐ended questions (*n* = 12) [22, 23, 24, 26, 27, 31, 39, 40, 41, 46, 48, 50], prospective observation (*n* = 2) [34, 53], retrospective observation (*n* = 2) [25, 33], and quality improvement methods (*n* = 1) [26].

Four key themes emerged from our review of the literature regarding the current state and quality of GOC discussions in surgical settings:

1. GOC discussions are reactive and initiated in response to imminent or acute changes in health status.
2. Surgeons enter GOC discussions with ambiguity around the limits of patient autonomy and a sense of duty to prioritize surgical treatment.
3. Objectives of GOC discussions are strongly related to the surgeon's perception of being the curator of information.
4. Outcomes of GOC discussions are determined by selecting from a set of standard treatment pathways rather than by establishing an understanding of patients' goals and values.

1. GOC discussions are reactive and initiated in response to imminent or acute changes in health status.

Surgeons initiated GOC discussions in response to acute changes in health status or progression of disease [22, 23, 26, 32, 38, 45, 49]. The initiation of GOC discussions reflected a reactive nature, as surgeons prioritized identifying current health status and formulating plans for short‐term management or “the next day”. They believed that patients who were acutely ill were most in need of having a GOC discussion [18, 45, 49]. As a result, most surgical trainees would primarily participate in GOC discussions in the ICU or inpatient setting in response to acute changes in health status [38].

In contrast, patients preferred having GOC discussions early on, prior to their prognosis worsening, with many preferring to engage in advance care planning even before receiving a diagnosis so they could minimize burdens on their families and gain a sense of control [24]. Patients also risk losing capacity in acute health events, thus initiating GOC discussions in response to acute health deteriorations may lead to missed opportunities to participate fully in a comprehensive discussion [26]. In one study on the frequency of end‐of‐life discussions, older patients perceived that they were more frequent compared with younger healthier patients [34]. This highlights surgeons' tendencies to initiate GOC discussions with sicker or deteriorating patients over more stable ones [34].

GOC discussions were also reactive in terms of patient readiness [30]. Surgeons often looked for cues indicating that a patient was ready to discuss GOC [30] such as follows: a patient asking about risks and complications or if patients were perceived to be emotionally well enough [23, 28, 35, 43]. Surgeons used an “eyeball” technique to assess patient's readiness for having a GOC discussion [46]. This involved determining whether a patient would be able to process large quantities of information quickly, evaluating frailty, waiting for patients to understand their disease, and judging their emotional state [19, 28, 29, 35]. If surgeons felt that patients were overwhelmed, discussions on GOC would be delayed [28, 47]. Although ensuring patient readiness for GOC discussions is important, relying on patient cues and waiting for acute changes in health to engage in GOC discussions can lead to patients feeling unprepared, unsupported, and blindsided when issues arise later in their care trajectory [44, 52].

2 Surgeons enter GOC discussions with ambiguity around the limits of patient autonomy and a sense of duty to prioritize surgical treatment.

When considering initiating GOC discussions—in particular around resuscitation, where certain patient preferences are not clinically feasible—surgeons felt ambiguous about the extent of patient autonomy [24, 29, 45]. Though surgeons emphasized the importance of patient autonomy, they remained skeptical about the extent of this autonomy in scenarios in which patients “don't really have choice”. For instance, surgeons described patients and families pushing for full cardiopulmonary resuscitation despite its' futility [24, 29, 45]. Surgeons highlighted the disconnection between patient preferences and clinical reality. This sometimes led to either hesitancy with initiating advance care planning and palliative care discussions or the focus being on documentation of GOC discussions [30, 31, 38]. Nonetheless surgeons recognized the value in understanding patients' perspectives on a meaningful life and their self‐determined risk‐to‐benefit analysis [29, 47].

Surgeons also considered their own comfort with leading GOC discussions before having such conversations. Surgical trainees and surgeons reported inadequate training in having discussions related to advance care planning, palliative, hospice, or end‐of‐life care, and thus lacked the confidence to facilitate them [24, 26, 27, 34, 41, 46, 50, 53]. Surgical trainees felt more comfortable listing and describing treatment options [41]. Many felt that lack of time was a significant barrier to having comprehensive discussions and were more likely to lead GOC discussions if they perceived that they would require less time to understand a patient's GOC [49]. Surgeons recognized that having effective GOC discussions required trust and familiarity [39, 47, 51]. They noted that patients may be better served by other clinicians with more time or established long‐term relationships—such as social work, nursing, or the patient's primary care physician [18, 19, 28, 38, 39, 47, 49].

Doubts around having GOC discussions extended beyond the above barrier. Some surgeons felt that it was not their unique responsibility, which ought to be shared [19]. This created role confusion as surgeons felt their main priority should be the surgical treatment [19, 20, 35, 38, 47]. Importantly, surgeons' commitment to treatment and cure also manifested as reluctance to acknowledge or discuss death, leading the tendency to believe that patients' main goal is “to be cured” [21, 24, 25, 48, 49].

3 Objectives of GOC discussions are strongly related to the surgeon's perception of being the curator of information.

Surgeons viewed GOC discussions as an opportunity to reinforce the “gravity of the surgical intervention itself” and to educate patients about the potential limitations or futility of surgery and postoperative life‐supporting therapy [18]. When surgeons felt that surgery was best, they used GOC discussions for instilling hope and motivating patients to buy‐in [19]. These discussions were viewed as opportunities to provide information about the illness and treatment and to provide a surgical opinion [29, 30, 35, 38]. GOC conversations were considered unidirectional, for patients to develop an understanding of the surgeons' expectations, during which eliciting patients' preferences and goals—including reduced pain, prolongation of life, or returning to daily activities—was not emphasized [45].

Both surgeons and patients expressed concerns that discussing advance directives or postoperative complications, such as feeding tubes and resuscitation, could distress patients and families. As a result, surgeons acted as curators of information carefully deciding when a GOC discussion was appropriate. To avoid overwhelming patients, surgeons focused on sharing key biomedical facts about the surgery and its purpose aiming to determine if patients wished to proceed [22].

4 Outcomes of GOC discussions are determined by selecting from a set of standard treatment pathways rather than by establishing an understanding of patients' goals and values.

Although surgeons acknowledged the importance of aligning treatments with a patient's goals [27, 42, 49], discussions often focused on management options [23]. Surgeons tended to focus on addressing immediate surgical issues, often asking patients to express a preference for specific treatments without delving into general goals or values [36, 37, 45].

Patients and their families nonetheless felt unprepared when serious complications occurred. Patients found it difficult to translate a list of discrete complications into an understanding of how their life would be affected [44, 52]. Patients reported that their surgeon did not present alternatives that were acceptable to them and that surgeons did not go beyond listing treatments and risks [54]. Paradoxically, although patients felt “blindsided” when complications occurred, they did not think that preoperative discussion of preferences for life‐sustaining treatments was important [23].

The focus on making specific care decisions was even more prevalent when family members were involved in the discussion [31]. Family members played a dominant role in end‐of‐life decision‐making and oftentimes, the patient's own priorities were omitted, especially when discussions did not include the patient due to loss of capacity [31].

Though the literature highlighted the surgeon's role in leading GOC conversations [26, 30, 45], certain topics would not be addressed unless introduced by the patient [51]. For instance, surgeons tended not to describe the process of dying nor mention pain or possible symptoms unless patients specifically asked [30]. This might be because surgeons and patients felt that discussing advance directives and graphically describing resuscitation and postoperative treatments of complications might be overwhelming for patients [22]. Thus, both patients and surgeons should be encouraged to ask about risks of surgery, future complications, to open the door to these conversations [22]. However, physicians also acknowledged that it should not be the sole responsibility of surgeons or patients to start conversations on advance care planning and code status [38, 53]. This highlighted the importance of teamwork and mutual accountability among members of the healthcare team [38, 53].

## 4 Discussion

In this paper, we identify key themes regarding the initiation, objectives, contents, and outcomes of GOC discussions in surgical settings. Hesitancy from surgeons to initiate GOC discussions often stems from difficulty reconciling patient autonomy with practical clinical considerations. This tension becomes more pronounced in discussions with patients and families regarding cardiopulmonary resuscitation and palliation. Hands‐on education and guidelines on balancing clinical opinion while honoring patient choice would be useful for surgeons who often face situations when patient goals conflict with the clinical reality. For example, the Best Case/Worst Case framework can be used by surgeons to communicate with patients about management options for serious illness [55]. The Best Case/Worst Case framework increases shared decision‐making and helps surgeons structure complex conversations to support patients and their families [55].

All studies were published in 2006 or later, suggesting that empirical research on the quality and content of GOC discussions in surgery is a relatively recent development. Few studies reported race and/or ethnicity data and specifically included participants of color. No studies reported on religion. There was a perceived lack of cultural competency exhibited during GOC discussions; however, these gaps were not specified [39, 51]. Surgeons noted patients with hearing or language barrier complicated communication [47, 51]. Further research is required to understand the experiences of GOC discussions for different groups to make GOC discussions culturally sensitive. Beliefs about health, autonomy, and death are deeply influence by cultural values; a one‐size‐fits‐all approach can lead to misunderstanding, mistrust, and care that does not reflect the patient's values. Preferences around family involvement in healthcare decision‐making may vary between cultures; it is important to approach these conversations with cultural humility and willingness to adapt one's communication style.

Most articles also reported on populations receiving or delivering surgical care in academic settings; it would be relevant to compare differences between academic and community settings including rural settings. Literature that focused on sharing the perspectives of surgeons and surgical trainees included populations that largely comprised of men; reflecting the fact that women have been underrepresented in surgical specialties [56, 57]. The lack of female representation in surgery can affect patient outcomes [58, 59, 60, 61]. Previous studies noted sex‐ and gender‐based differences in communication styles, practices, and patient–provider relationships, such that female physicians engage in more patient‐centered communication compared with their male colleagues [58, 59, 60, 61]. Communication techniques that are more frequently observed in female physicians have been linked to higher patient satisfaction, improved health outcomes, and lower postoperative complication rates [58, 61].

Patients felt “blindsided” when complications occurred, citing that although their surgeon discussed risks of surgery, it was difficult to translate this list of discrete complications into an understanding of how their life would be impacted. Despite these feelings, patients still did not feel it was necessary to have preoperative discussions about their preferences for life‐sustaining treatment. Further research is needed to understand patient hesitancy to discuss life‐sustaining treatments preoperatively and develop strategies for having these conversations in a way that is concordant with patients and their families' wellbeing. The American POLST (Physician Orders for Life‐Sustaining Treatment) form is an advance care planning tool used to facilitate conversations around cardiopulmonary resuscitation, medical interventions, and nutrition [62]. Such tools can help to introduce difficult conversations about life‐sustaining treatment, which patients and physicians have found valuable for strengthening patient autonomy [62].

In this paper, we identified key factors that influence the initiation, objectives, and contents of GOC discussions in surgery. Evidently, numerous barriers exist in terms of having effective GOC discussions; however questions remain regarding ways to address these barriers and what constitutes a successful GOC discussion. Although initiating GOC discussions in response to acute health changes has negative consequences, doing so may be common practice among surgeons because patients appear “more ready” to have difficult conversations at these points. Defining optimal timing or equipping surgeons with effective tools and training to accurately appreciate patient readiness for GOC discussions would be beneficial. Jackson et al. provide a stepwise approach for assessing patient readiness and cultivating prognostic awareness, which begins a self‐assessment question that offers insight on the patient's understanding of their illness and willingness to discuss the future [63]. Beginning with asking patients “what is your sense of how you are doing” provides clinicians with the opportunity to respond to patients' cues and shape GOC discussions accordingly [63].

Surgeons would also benefit from more education on patient autonomy and how to honor this in practice. The ambiguity and skepticism which surgeons feel about the extent of patient autonomy especially when patient preferences are not clinically feasible may stem from lack of surgeons' understanding regarding autonomy. Patient autonomy is defined as the right of patients to make decisions about their care free from coercion or external control [64, 65]. Honoring patient autonomy does not necessarily mean that the patient's preference for their care is the final decision. Rather, honoring patient autonomy involves a process by which clinicians provide critical education about management options and assist patients in making a decision that aligns with their personal values [64, 65]. Education for clinicians regarding patient autonomy begin in medical school. Patient participation in the development and delivery of medical undergraduate education fosters a deeper appreciation among medical students for patient‐centered care [66]. Incorporating patients as teachers provides medical students with opportunities to develop collaborative communication skills while learning to balance clinical expertise with patient empowerment [66].

Surgical practice and education should recognize the importance of a culture that prioritizes effective GOC discussions. Systemic changes in the workplace may be necessary to prioritize such discussions. Team‐based practices should be explored further. Support from leadership and all care team members is essential to ensure the appropriate time, space, and practical learning opportunities for robust GOC discussions to occur. Organizational role theory examines how one's behavior in an organization is shaped by expectations associated with their role [67]. Ma et al. use organizational role theory to describe how the prioritization of GOC conversations requires role expansion; we must set clear expectations across disciplines around GOC conversation related tasks and instill collective responsibility for taking on these discussions [67].

### 4.1 Limitations

Scoping reviews are inherently limited by their focus on providing breadth rather than depth of information on a specific topic. As such, we did not conduct a meta‐analysis. However, we felt that conducting a scoping review for this topic was appropriate, given that our objective was to capture the current landscape of literature on the quality of goals of care discussions in surgical settings. Our review is also limited to only studies disseminated in English and we did not include articles discussing pediatric populations.

### 4.2 Conclusion

This scoping review examined 37 studies on the current state and quality of GOC discussions in surgical settings, highlighting several key themes around the reactive nature of GOC discussions, perceived ambiguity around the extent of patient autonomy, and surgeons' perspectives on their role in leading GOC discussions. We highlight the critical need for improving the quality and timing of GOC discussions in surgery. These findings have practical implications for surgical trainees, who can benefit from communications training, and medical educators, who may design curricula that normalize GOC conversations as a core component of surgical training. Further research should explore patient perspectives on improving GOC discussions in surgery and expand the diversity of reported experiences to include patients from diverse ethnic background and genders and individuals from rural and lower‐resource communities. Further exploration will advance our understanding of how to improve the quality of GOC discussions in surgery, ensuring that all receive patient‐centered and goal‐concordant care.

## Author Contributions

**Amanda Mac:** formal analysis, validation, writing – original draft, writing – review and editing. **Yerin Lee:** formal analysis, validation, writing – review and editing. **Selena Zhang:** formal analysis, validation, writing – review and editing. **Abu Sadat Mohammad Nurunnabi:** validation, writing – review and editing. **Marina Englesakis:** methodology, data curation, writing – review and editing. **Karen Devon:** conceptualization, formal analysis, validation, project administration, writing – review and editing.

## Ethics Statement

The authors have nothing to report.

## Conflicts of Interest

The authors declare no conflicts of interest.

## Supporting information


Suporting Information S1

## Data Availability

The authors have nothing to report.
