# Supplementary material for: A Scoping Review on Goals of Care Discussions in Surgery: How Are We Doing and How Can We Do Better?
Source: World J Surg. 2025 Aug 23;49(10):2828–36. doi: 10.1002/wjs.70070 (PMC12515031; doi:10.1002/wjs.70070)
Supplement: Supplementary file 1 — Suporting Information S1 [file WJS-49-2828-s001.docx]

Supplement 1: MEDLINE Search Strategy

Ovid MEDLINE(R) 1946 to July 28, 2022

| **#** | **Searches** |
| --- | --- |
| 1 | Advance Directives/ |
| 2 | exp Advance Care Planning/ |
| 3 | Euthanasia, Passive/ |
| 4 | Living Wills/ |
| 5 | Medical Futility/ |
| 6 | Resuscitation Orders/ |
| 7 | Right To Die/ |
| 8 | Therapeutic Misconception/ |
| 9 | Treatment Refusal/ |
| 10 | Withholding Treatment/ |
| 11 | "advance decision".mp. [ Term used in the UK ] |
| 12 | "advance* health care plan*".mp. |
| 13 | "advance* healthcare plan*".mp. |
| 14 | "advance* medical direct*".mp. |
| 15 | "advance* medical plan*".mp. |
| 16 | "advance? care plan*".mp. |
| 17 | "advance? directive?".mp. |
| 18 | "care preference?".mp. |
| 19 | "DNR/DNI".ti,ab. |
| 20 | "do not intubate".mp. |
| 21 | "do not resuscitate".mp. |
| 22 | "end of life directive?".mp. |
| 23 | "end of life plan*4".mp. |
| 24 | "health care prox*".mp. |
| 25 | "healthcare prox*".mp. |
| 26 | "living will?".mp. |
| 27 | "power? of attorney".mp. |
| 28 | "resuscitat* order?".mp. |
| 29 | (discuss* adj2 care).mp. |
| 30 | ((DNI or DNIs) and (resus* or intub*)).ti,ab. |
| 31 | ((DNR or DNRs) and (resus* or intub*)).ti,ab. |
| 32 | (advance? adj1 directive?).mp. |
| 33 | (futil* adj2 treatment?).mp. |
| 34 | (goal* adj2 concord* adj2 care).mp. |
| 35 | (life adj2 limit* adj2 ill*).mp. |
| 36 | (life adj2 shorten* adj2 decision*).mp. |
| 37 | (life-prolonging adj2 (therap* or treatment)).mp. |
| 38 | (life-prolonging adj2 decision*).mp. |
| 39 | (life adj2 sustain* adj2 (therap* or treatment*)).mp. |
| 40 | (medical* adj1 aid??? adj2 dying).mp. |
| 41 | (medical* adj1 assist* adj2 dying).mp. |
| 42 | (medical* adj2 futil*).mp. |
| 43 | (nonbenefi* adj2 (surg* or therap* or treatment*)).mp. |
| 44 | (non-benefi* adj2 (surg* or therap* or treatment*)).mp. |
| 45 | (refus* adj2 treatment*).mp. |
| 46 | (surgical* adj2 futil*).mp. |
| 47 | (withdraw* adj2 care).mp. |
| 48 | (withdraw* adj2 therapy).mp. |
| 49 | (withdraw* adj2 treatment).mp. |
| 50 | (code status and (decid* or decision* or plan or plans or planning or care or order?)).mp. |
| 51 | goal? of care.mp. |
| 52 | ("goal? of surgery" and (decid* or decision* or plan or plans or planning or care or order?)).mp. |
| 53 | ("goal? of therapy" and (decid* or decision* or plan or plans or planning or care or order?)).mp. |
| 54 | ("goal? of treatment" and (decid* or decision* or plan or plans or planning or care or order?)).mp. |
| 55 | or/1-54 [ Advance Directives or Advance Care Planning or Goals of Care & related terms ] |
| 56 | "Delayed Emergence from Anesthesia"/ |
| 57 | Anesthesia Recovery Period/ |
| 58 | co.xs. and post*.mp. [Complications exploded floating subheading] |
| 59 | exp "Anesthesia and Analgesia"/ |
| 60 | exp Intraoperative Complications/ |
| 61 | exp Intraoperative Period/ |
| 62 | exp Postoperative Care/ |
| 63 | exp Postoperative Complications/ |
| 64 | exp Postoperative Period/ |
| 65 | exp Specialties, Surgical/ |
| 66 | exp Surgeons/ |
| 67 | exp Surgical Procedures, Operative/ |
| 68 | Operating Room Nursing/ |
| 69 | Operating Rooms/ |
| 70 | Operative Blood Salvage/ |
| 71 | Operative Time/ |
| 72 | Perioperative Care/ |
| 73 | Perioperative Medicine/ |
| 74 | Perioperative Nursing/ |
| 75 | Perioperative Period/ |
| 76 | Postanesthesia Nursing/ |
| 77 | Postoperative Cognitive Complications/ |
| 78 | Reoperation/ |
| 79 | Salvage Therapy/ |
| 80 | su.fs. [ Surgery floating subheading ] |
| 81 | tr.fs. [ Transplant floating subheading ] |
| 82 | (after adj6 (operation? or operative??)).mp,kw. |
| 83 | (after adj6 (surgery or surgeries or surgical*)).mp,kw. |
| 84 | (aneurysm* adj2 repair*).mp,kw. |
| 85 | (cardiac adj2 catheter* adj2 ablat*).mp,kw. |
| 86 | (carotid adj2 endarterectom*3).mp,kw. |
| 87 | (consequen* adj6 (surgery or surgeries or surgical*)).mp,kw. |
| 88 | (coronary adj2 catheter* adj2 ablat*).mp,kw. |
| 89 | (coronary adj2 radiofrequenc* adj2 ablat*).mp,kw. |
| 90 | (coronary adj3 bypass*).mp,kw. |
| 91 | (coronary adj3 catheteri*).mp,kw. |
| 92 | (follow* adj6 (operation? or operative??)).mp,kw. |
| 93 | (follow* adj6 (surgery or surgeries or surgical*)).mp,kw. |
| 94 | (heart adj2 radiofrequenc* adj2 ablat*).mp,kw. |
| 95 | (heart adj2 valve? adj2 repair*).mp,kw. |
| 96 | (heart adj2 valve? adj2 replac*).mp,kw. |
| 97 | (hybrid-maze adj2 procedure*).mp,kw. |
| 98 | (mitral valve? adj2 repair*).mp,kw. |
| 99 | (mitral valve? adj2 reparat*).mp,kw. |
| 100 | (near adj2 miss*).mp,kw. |
| 101 | (open adj1 reduction?).mp,kw. |
| 102 | (perioperat* or peri-operat*).mp,kw. |
| 103 | (readmi* or re-admi*).mp,kw. |
| 104 | (respiratory adj2 aspirat*).mp,kw. |
| 105 | (subsequent* adj6 (operation? or operative??)).mp,kw. |
| 106 | (subsequent* adj6 (surgery or surgeries or surgical*)).mp,kw. |
| 107 | (transmyocardia* adj3 revasculari*).mp,kw. |
| 108 | (transmyocardia* adj5 ablat*).mp,kw. |
| 109 | adenectom*.mp,kw. |
| 110 | adrenalectom*.mp,kw. |
| 111 | an?esthe*.mp,jw,in. |
| 112 | angioplast*3.mp,kw. |
| 113 | appendectom*.mp,kw. |
| 114 | arthrectom*.mp,kw. |
| 115 | arthroplast???.mp,kw. |
| 116 | atherectom*3.mp,kw. |
| 117 | CABG.mp,kw. |
| 118 | caesarean*.mp,kw. |
| 119 | cardiac ablat*.mp,kw. |
| 120 | cardiomyoplast*3.mp,kw. |
| 121 | cervicectom*.mp,kw. |
| 122 | cesarean*.mp,kw. |
| 123 | cholecystectom*.mp,kw. |
| 124 | colectom*.mp,kw. |
| 125 | coronary artery bypass surger*.mp,kw. |
| 126 | cystectom*.mp,kw. |
| 127 | cytokine release syndrome?.mp,kw. |
| 128 | delayed emergence.mp,kw. |
| 129 | discectom*.mp,kw. |
| 130 | diskectom*.mp,kw. |
| 131 | duodenectom*.mp,kw. |
| 132 | emergence deliri???.mp,kw. |
| 133 | esophagectom*.mp,kw. |
| 134 | fundectom*.mp,kw. |
| 135 | gastrectom*.mp,kw. |
| 136 | gerionco*.mp,kw. |
| 137 | glossectom*.mp,kw. |
| 138 | gonadectom*.mp,kw. |
| 139 | hemiarthroplast???.mp,kw. |
| 140 | hemi-arthroplast???.mp,kw. |
| 141 | hemicolectom*.mp,kw. |
| 142 | hemi-colectom*.mp,kw. |
| 143 | hepatectom*.mp,kw. |
| 144 | hypophysectom*.mp,kw. |
| 145 | iridectom*.mp,kw. |
| 146 | jejunectom*.mp,kw. |
| 147 | laparoscop*.mp,kw. |
| 148 | laparotom*.mp,kw. |
| 149 | laryngectom*.mp,kw. |
| 150 | lobectom*.mp,kw. |
| 151 | lumpectom*.mp,kw. |
| 152 | lymphadenectom*.mp,kw. |
| 153 | lymph-adenectom*.mp,kw. |
| 154 | lymphectom*.mp,kw. |
| 155 | mandibulectom*.mp,kw. |
| 156 | mastectom*.mp,kw. |
| 157 | mastoidectom*.mp,kw. |
| 158 | maxillectom*.mp,kw. |
| 159 | mesohepatectom*.mp,kw. |
| 160 | meso-hepatectom*.mp,kw. |
| 161 | metastasectom*.mp,kw. |
| 162 | MIDCAB.mp,kw. |
| 163 | myectom*.mp,kw. |
| 164 | myomectom*.mp,kw. |
| 165 | necrosectom*.mp,kw. |
| 166 | nephrectom*.mp,kw. |
| 167 | neurosurg*.mp,kw. |
| 168 | obstetr*.mp,in,kw,kf. |
| 169 | obstetr*.mp,kw. |
| 170 | oesophagectom*.mp,kw. |
| 171 | oncosurg*.mp,kw. |
| 172 | onco-surg*.mp,kw. |
| 173 | oncosurg*.mp. |
| 174 | onco-surg*.mp. |
| 175 | oophorectom*.mp,kw. |
| 176 | operati*.mp,kw. |
| 177 | orchidectom*.mp,kw. |
| 178 | orchiectom*.mp,kw. |
| 179 | pancreatectom*.mp,kw. |
| 180 | pancreaticoduodenectom*.mp,kw. |
| 181 | pancreatico-duodenectom*.mp,kw. |
| 182 | parathyroidectom*.mp,kw. |
| 183 | para-thyroidectom*.mp,kw. |
| 184 | peroperativ*.mp,kw. |
| 185 | pharyng*esophagectom*.mp,kw. |
| 186 | pharyngectom*.mp,kw. |
| 187 | pharyngolaryngoesophagectom*.mp,kw. |
| 188 | pneumonectom*.mp,kw. |
| 189 | post*an?esth*.mp,kw. |
| 190 | post-*an?esth*.mp,kw. |
| 191 | post*cardiac.mp,kw. |
| 192 | post*ectom*.mp,kw. |
| 193 | post-*ectom*.mp,kw. |
| 194 | post*laparoscop*.mp,kw. |
| 195 | post-*laparoscop*.mp,kw. |
| 196 | post*laparotom*.mp,kw. |
| 197 | post-*laparotom*.mp,kw. |
| 198 | post*microsurg*.mp,kw. |
| 199 | post-*microsurg*.mp,kw. |
| 200 | post-*operat*.mp,kw. |
| 201 | post*ostom*.mp,kw. |
| 202 | post-*otom*.mp,kw. |
| 203 | post*pericardiot*.mp,kw. |
| 204 | post*pex*.mp,kw. |
| 205 | post-*pex*.mp,kw. |
| 206 | post*plast*.mp,kw. |
| 207 | post-*plast*.mp,kw. |
| 208 | post-*procedur*.mp,kw. |
| 209 | post*reconstruct*.mp,kw. |
| 210 | post-*reconstruct*.mp,kw. |
| 211 | post*scop*.mp,kw. |
| 212 | post-*scop*.mp,kw. |
| 213 | post-*surg*.mp,kw. |
| 214 | post*surger*.mp,kw. |
| 215 | post-*surger*.mp,kw. |
| 216 | post*transplant*.mp,kw. |
| 217 | post-*transplant*.mp,kw. |
| 218 | postintervention*.mp,kw. |
| 219 | post-intervention*.mp,kw. |
| 220 | postoperat*.mp,kw. |
| 221 | post-operat*.mp,kw. |
| 222 | postpericardio*.mp,kw. |
| 223 | post-pericardio*.mp,kw. |
| 224 | postproced*.mp,kw. |
| 225 | postprocedur*.mp,kw. |
| 226 | post-procedur*.mp,kw. |
| 227 | postsurg*.mp,kw. |
| 228 | post-surg*.mp,kw. |
| 229 | proctocolectom*.mp,kw. |
| 230 | prostatect*.mp,kw. |
| 231 | quadrantectom*.mp,kw. |
| 232 | re-*operat*.mp,kw. |
| 233 | reoperat*.mp,kw. |
| 234 | re-operat*.mp,kw. |
| 235 | reresect*.mp,kw. |
| 236 | resect*.mp,kw. |
| 237 | rhinectom*.mp,kw. |
| 238 | salpingectom*.mp,kw. |
| 239 | salpingo-oophorectom*.mp,kw. |
| 240 | second look*.mp,kw. |
| 241 | second-look*.mp. |
| 242 | segmentectom*.mp,kw. |
| 243 | splenectom*.mp,kw. |
| 244 | subsegmentectom*.mp,kw. |
| 245 | sub-segmentectom*.mp,kw. |
| 246 | surgeon*.mp,kw. |
| 247 | surger*.mp,kw. |
| 248 | surger???.mp,kw. |
| 249 | surgical*.mp,kw. |
| 250 | takeback?.mp. |
| 251 | take-back?.mp. |
| 252 | thymectom*.mp,kw. |
| 253 | thyroidectom*.mp,kw. |
| 254 | tonsillectom*.mp,kw. |
| 255 | transfusion*.mp,kw. |
| 256 | transplant*.mp,kw. |
| 257 | trisegmentectom*.mp,kw. |
| 258 | tri-segmentectom*.mp,kw. |
| 259 | tumorectom*.mp,kw. |
| 260 | uretectom*.mp,kw. |
| 261 | uvulectom*.mp,kw. |
| 262 | vaginectom*.mp,kw. |
| 263 | vulvectom*.mp,kw. |
| 264 | or/56-263 [ Surgery or Perioperative Care ] |
| 265 | 55 and 264 [ Advance Directives/ACPs/Goals of Care + Surgery/Periop ] |
| 266 | Algorithms/ |
| 267 | Clinical Protocols/ |
| 268 | Critical Pathways/ |
| 269 | Decision Support Techniques/ |
| 270 | Decision Theory/ |
| 271 | exp *Disease Management/ |
| 272 | exp *Early Diagnosis/ |
| 273 | "*Early Detection of Cancer"/ |
| 274 | exp Algorithms/ |
| 275 | exp Benchmarking/ |
| 276 | exp Decision Trees/ |
| 277 | exp Guideline/ |
| 278 | exp Guidelines As Topic/ |
| 279 | exp Practice Guideline/ |
| 280 | exp Practice Guidelines As Topic/ |
| 281 | Guideline Adherence/ |
| 282 | Health planning guidelines/ |
| 283 | Patient Care Bundles/ |
| 284 | Physician's Practice Patterns/ |
| 285 | ((comply or complies or compliant or compliance) adj2 (policy or policies)).mp. |
| 286 | ((comply or complies or compliant or compliance) adj2 guideline*).mp. |
| 287 | ((comply or complies or compliant or compliance) adj2 protocol*).mp. |
| 288 | ((multimodal or multi-modal) adj2 recover*).mp. |
| 289 | ((multimodal or multi-modal) adj2 rehab*).mp. |
| 290 | (accelerat* adj2 care).mp. |
| 291 | (accelerat* adj2 recover*).mp. |
| 292 | (best adj practi#e?).mp. |
| 293 | (care adj bundle?).mp. |
| 294 | (care adj2 (path or paths or pathway or pathways)).mp. |
| 295 | (care adj2 map*).mp. |
| 296 | (care adj2 plan*).mp. |
| 297 | (clinical adj2 (path or paths or pathway or pathways)).mp. |
| 298 | (clinical adj2 protocol?).mp. |
| 299 | (critical adj2 (path or paths or pathway or pathways)).mp. |
| 300 | (decision adj2 tree?).mp. |
| 301 | (decision? adj2 aid?).mp. |
| 302 | (decision? adj2 analy*).mp. |
| 303 | (decision? adj2 model*).mp. |
| 304 | (decision? adj2 techni*).mp. |
| 305 | (enhanced adj2 recover*).mp. |
| 306 | (expedit* adj2 recover*).mp. |
| 307 | (fasttrack* or fast-track*).mp. |
| 308 | (gold?? adj1 standard?).mp. |
| 309 | (guidance adj2 (introduc* or issu* or impact* or effect* or disseminat* or distribut* or implement*)).mp. |
| 310 | (guideline or guidelines).mp. |
| 311 | (hasten* adj2 recover*).mp. |
| 312 | (lean adj2 pathway?).mp. |
| 313 | (management adj2 protocol*).mp. |
| 314 | (nurs* adj2 protocol*).mp. |
| 315 | (policy or policies).tw. |
| 316 | (practi#e adj1 parameter?).mp. |
| 317 | (practi#e adj1 pattern?).tw. |
| 318 | (practi?e adj2 (protocol* or policy or policies or guideline*)).mp. |
| 319 | (process?? adj2 (chart? or diagram* or flowchart*)).mp. |
| 320 | (recover* adj2 pathway*).mp. |
| 321 | (rule or rules).tw. |
| 322 | (standard? adj2 practi#e?).mp. |
| 323 | (treat* adj2 protocol?).mp. |
| 324 | (treatment adj2 plan*).mp. |
| 325 | (treatment adj2 protocol*).mp. |
| 326 | (treatment* adj2 (path or paths or pathway or pathways)).mp. |
| 327 | algorhythm*.mp. |
| 328 | algorism*.mp. |
| 329 | algorithm*.mp. |
| 330 | bench mark*.mp. |
| 331 | benchmark*.mp. |
| 332 | best practi*.mp. |
| 333 | decision tree?.mp. |
| 334 | flow chart?.mp. |
| 335 | flow diagram???.mp. |
| 336 | flowchart?.mp. |
| 337 | or/266-336 [ Algorithms or Best Practices or Care Pathways or Clinical Pathways or Critical Pathways ] |
| 338 | "Facility Regulation And Control"/ |
| 339 | "Joint Commission On Accreditation Of Healthcare Organizations"/ |
| 340 | Clinical Competence/ |
| 341 | Ed.fs. [Education Floating Subheading] |
| 342 | Education, Medical, Continuing/ |
| 343 | exp Academic Medical Centers/ |
| 344 | exp Accreditation/ |
| 345 | exp Certification/ |
| 346 | exp Communication/ |
| 347 | exp Communication Barriers/ |
| 348 | exp Competency-Based Education/ |
| 349 | exp Consensus Development Conference/ |
| 350 | exp Consensus Development Conferences As Topic/ |
| 351 | exp Consensus/ |
| 352 | exp Credentialing/ |
| 353 | exp Curriculum/ |
| 354 | exp Education, Medical, Graduate/ |
| 355 | exp Education, Medical/ |
| 356 | exp Education, Professional/ |
| 357 | exp Educational Measurement/ |
| 358 | exp Faculty, Medical/ |
| 359 | exp Faculty/ |
| 360 | exp Hospitals, Teaching/ |
| 361 | exp Inservice Training/ |
| 362 | exp Interdisciplinary Studies/ |
| 363 | exp International Educational Exchange/ |
| 364 | exp Licensure/ |
| 365 | exp Mentors/ |
| 366 | exp Models, Educational/ |
| 367 | exp Preceptorship/ |
| 368 | exp Professional Competence/ |
| 369 | exp Schools, Medical/ |
| 370 | exp Teaching Materials/ |
| 371 | exp Teaching/ |
| 372 | Hospitals, Teaching/ |
| 373 | Hospitals, University/ |
| 374 | Inservice Training/ |
| 375 | Pilot Projects/ |
| 376 | Professional Competence/ |
| 377 | Program Development/ |
| 378 | Program Evaluation/ |
| 379 | Specialty Boards/ |
| 380 | Training Support/ |
| 381 | (fellow or fellows or fellowship*).mp,kw. |
| 382 | (skill or skilled or skills).mp,kw. |
| 383 | (training? or trainee?).mp,kw. |
| 384 | accredit*.mp,kw. |
| 385 | academ*.mp,kw. |
| 386 | certif*.mp,kw. |
| 387 | competen*.mp,kw. |
| 388 | credential*.mp,kw. |
| 389 | curricul*.mp,kw. |
| 390 | curricula?.mp,kw. |
| 391 | educat*.mp,kw. |
| 392 | governance.mp,kw. |
| 393 | instruction.mp,kw. |
| 394 | learn*.mp,kw. |
| 395 | licenc*.mp,kw. |
| 396 | licens*.mp,kw. |
| 397 | mentor*.mp,kw. |
| 398 | outline?.mp,kw. |
| 399 | postgrad*.mp,kw. |
| 400 | post-grad*.mp,kw. |
| 401 | preceptor*.mp,kw. |
| 402 | proctor*.mp,kw. |
| 403 | (program? or programme?).mp,kw. |
| 404 | qualif*.mp,kw. |
| 405 | seminar?.mp,kw. |
| 406 | standard?.ti,ab. |
| 407 | syllab*.mp,kw. |
| 408 | train*.mp,kw. |
| 409 | workshop*.mp,kw. |
| 410 | intervention?.ab. /freq=2 or intervention?.ti. |
| 411 | or/338-410 [ Education & related terms ] |
| 412 | 337 or 411 [ Best Practices OR Education ] |
| 413 | 265 and 412 [ Advance Directives/ACPs/Goals of Care + Surgery/Periop + Best Practices/Education ] |
| 414 | limit 413 to english language |
| 415 | 414 not (exp animals/ not (exp animals/ and (exp humans/ or exp patients/))) |
| 416 | limit 414 to humans |
| 417 | 415 or 416 |
| 418 | 417 not ((study guide or preprint).pt. or (arxiv or medrxiv or biorxiv or research square).so. or (AHRQ* or StatPearls or Genereviewsovidsup).bt. or chapter.pr. or NB*.bk.) |
| 419 | remove duplicates from 418 [ removal of internal database duplicate citations ] |
| 420 | 419 [ Advance Directives/ACPs/Goals of Care + Surgery/Periop + Best Practices/Education; limits and update period applied ] |

Supplement 2. Summary of included articles.

| **Study** | **Location, Participants** | **Purpose, Design, Single vs. Multi-Centre, Timeframe** | **Main findings** |
| --- | --- | --- | --- |
| 1. Bergenholtz et al., 2019 [18] | **Country of publication:** Denmark  **Number and type of participants:** 14 physicians  **Proportion of participants that were surgical (surgical patients, trainees or surgeons):** 50%  **Age:** range of 20 to 59  **Ratio of females to males:** 6 to 1  **Race/ethnicity:** not recorded  **Setting of surgery:** elective or scheduled oncology  **Type of surgery:** oncology  **Academic or community:** not recorded | **Purpose:** Explored existing practices regarding end-of-life conversations in an acute care hospital.  **Design:** Qualitative study. Observed conversations between patients, doctors and nurses. Focus group interviews with doctors and nurses.  **Single or multi-centre:** single | End-of-life conversations were part of daily clinical practice. However, physicians felt they did not have adequate training, role clarity, time and physical space to facilitate these conversations. Conversations were initiated by changes in health status (progressive and more acute disease requiring a plan for the near future) and intuition of the clinician (waiting for the patient to be open to having this discussion, courage of the clinician). |
| 2. Bonanno et al., 2019 [19] | **Country of publication:** USA  **Number and type of participants:** 52 residents, 38 staff  **Proportion of participants that were surgical (surgical patients, trainees or surgeons):** 100%  **Age:** not recorded  **Ratio of females to males:** not recorded  **Race/ethnicity:** not recorded  **Setting of surgery:** not recorded  **Type of surgery:** not recorded  **Academic or community:** academic | **Purpose:** Described the palliative care educational needs of general surgery trainees.  **Design:** survey  **Single or multi-centre:** multi | Residents often led GOC conferences in the Intensive Care Unit (ICU), while staff tended to facilitate these conversations in ward and clinic. Residents felt they had limited outpatient clinic exposure and palliative care teaching in the elective preoperative setting. Residents noted that the main barrier to consultation with palliative care was attending preference. |
| 3. Bradley et al., 2010 [20] | **Country of publication:** USA  **Number and type of participants:** 10 physicians  **Proportion of participants that were surgical (surgical patients or surgeons):** not recorded (“subjects were primarily surgeons”)  **Age:** not recorded  **Ratio of females to males:** 8 to 2  **Race/ethnicity:** not recorded  **Setting of surgery:** not recorded  **Type of surgery:** 10% trauma and surgical critical care, 10% transplant, 10% neurosurgery, 10% cardiac surgery, 20% surgical oncology, 10% vascular surgery, 10% cardiac anesthesia, 10% internal medicine/preoperative clearance, 10% general vascular surgery  **Academic or community:** 10% private practice, 90% academic | **Purpose:** Explored physicians’ opinions of advance directives for surgical patients.  **Design:** Qualitative study. 1:1 interview.  **Single or multi-centre:** multi | Advance directives serve as a platform for discussing the limitations of surgery and life-supporting therapy. Advance directives should be addressed to reinforce the gravity of the surgical intervention itself. However, physicians were frustrated with balancing their drive for surgical cure and the treatment limitations posed by advance directives (Eg. being an advocate for aggressive surgical care vs. simultaneously counselling on end-of-life decisions; operative goals conflicting with the patient’s overarching goals of care). Physicians were concerned that discussing advance directives with patients preoperatively might create unnecessary apprehension for the patient when deciding whether to consent to a procedure. Some felt that everyone should have an advance directive while others felt only certain subgroups should (patients with severe comorbidities or cancer). |
| 4. Chikada et al., 2021 [21] | **Country of publication:** Japan  **Number and type of participants:** 57 family members of deceased patients  **Proportion of participants that were surgical (surgical patients, trainees or surgeons):** 100%  **Age:** median of 68 (range of 35 to 90)  **Ratio of females to males:** 41 to 16  **Race/ethnicity:** Japanese  **Setting of surgery:** elective or scheduled oncology  **Type of surgery:** high-grade glioma  **Academic or community:** academic | **Purpose:** Determined the appropriateness of timing and the extent of patient involvement in end-of-life discussions, and their impact on high-grade glioma patients.  **Design:** survey  **Single or multi-centre:** single | 91% of family members participated in end-of-life discussions. 47% of discussions involved family only, without the patient present. 26% of those who had family-only involvement cited that they did not want the patient to become aware of the details of their medical condition. Patient goals and priorities were discussed in 28% of conversations. |
| 5. Cooper et al., 2016 [22] | **Country of publication:** USA  **Number and type of participants:** 23 physicians  **Proportion of participants that were surgical (surgical patients, trainees or surgeons):** 43.4%  **Age:** not recorded  **Ratio of females to males:** not recorded  **Race/ethnicity:** not recorded  **Setting of surgery:** not recorded  **Type of surgery:** 50% acute care surgery, 10% general surgery, 10% vascular surgery, 30% surgical oncology  **Academic or community:** academic | **Purpose:** Established key elements of a communication framework to support surgeons, patients with serious illness and their families in decisions about surgery in the acute setting.  **Design:** Qualitative study. Panel discussion.  **Single or multi-centre:** multi | Key elements for effective communication include: formulating a prognosis for the patient with and without surgery, eliciting the patient’s understanding of their illness, informing about the life-threatening nature of their condition and potential for life support or loss of independence, presenting all options including potential postoperative trajectories and palliative approaches, understanding what is acceptable to the patient regarding life-extending vs comfort-focused care, recommending treatments in the context of the patient’s illness and goals, and supporting the decision of the patient. |
| 6. Cunningham et al., 2018 [23] | **Country of publication:** USA  **Number and type of participants:** 289 physicians  **Proportion of participants that were surgical (surgical patients, trainees or surgeons):** 15.5%  **Age:** mean of 46.84, standard deviation of 11.67  **Ratio of females to males:** 17 to 22  **Race/ethnicity:** not recorded  **Setting of surgery:** emergency benign or oncology  **Type of surgery:** burn surgery  **Academic or community:** 77.8% academic, 11.1% community, 11.1% other | **Purpose:** Explored perspectives of burn surgeons and palliative care specialists regarding the importance of GOC conversations for burned seniors, confidence in their own specialty’s ability to conduct these conversations, and confidence in the ability of the other specialty to do so.  **Design:** survey  **Single or multi-centre:** multi | Both palliative care physicians and burn surgeons equally agreed regarding the importance of GOC discussions with injured geriatric patients. Both specialties favoured their own specialty in leading the discussion. However, burn surgeons were less comfortable holding GOC discussions alone. Both agreed that discussions should occur within 72 hours of admission. |
| 7. Dalvin et al., 2018 [24] | **Country of publication:** USA  **Number and type of participants:** 8 medical students, 11 residents, 1 staff  **Proportion of participants that were surgical (surgical patients, trainees or surgeons):** 60%  **Age:** not recorded  **Ratio of females to males:** not recorded  **Race/ethnicity:** not recorded  **Setting of surgery:** not recorded  **Type of surgery:** general surgery  **Academic or community:** academic | **Purpose:** Evaluated the baseline knowledge and test the effectiveness of an educational session for surgical residents focused on palliative care, hospice care and advance care planning.  **Design:** survey  **Single or multi-centre:** single | Pretest results ranged from 29% to 79% correct, with a mean score of 60%. 18% of residents had previous training in palliative care, hospice care, advance care planning, and end of life ethics. 60% of participants had previous education around these topics in medical school. |
| 8. Demyan et al., 2022 [25] | **Country of publication:** USA  **Number and type of participants:** 10 physicians  **Proportion of participants that were surgical (surgical patients, trainees or surgeons):** 100%  **Age:** not recorded  **Ratio of females to males:** 4 to 6  **Race/ethnicity:** not recorded  **Setting of surgery:** elective or scheduled oncology  **Type of surgery:** pancreatic cancer  **Academic or community:** 80% academic | **Purpose:** Evaluated surgeons’ insights, perceptions and biases regarding preoperative advance care planning.  **Design:** Qualitative study. 1:1 interview.  **Single or multi-centre:** multi | 80% emphasized importance of offering hope, enthusiasm and motivation. All reported that lack of time is the most significant barrier to building rapport and discussing patients’ long-term goals of care. 30% felt strongly that advance care planning should not occur before surgery and 70% said it should occur as a point on a timeline of disease progression, when death is imminent. Surgeons recognized that there is sometimes misalignment between surgeon and patient goals, however the main job of the surgeon is to remove the tumour. |
| 9. El-Sahwi et al., 2012 [26] | **Country of publication:** USA  **Number and type of participants:** 142 physicians  **Proportion of participants that were surgical (surgical patients, trainees or surgeons):** 100%  **Age:** range of 30 to 60  **Ratio of females to males:** 57 to 85  **Race/ethnicity:** not recorded  **Setting of surgery:** elective or scheduled oncology  **Type of surgery:** gynecologic oncology  **Academic or community:** not recorded | **Purpose:** Identified practices and attitudes of gynecologic oncologists regarding end-of-life discussions.  **Design:** survey  **Single or multi-centre:** multi | >50% deferred end-of-life discussions until the patient sustained a major change in functional or medical status. 43% characterized an end-of-life discussion as an ongoing process. |
| 10. Eli et al., 2022 [27] | **Country of publication:** United Kingdom  **Number and type of participants:** 34 physicians  **Proportion of participants that were surgical (surgical patients, trainees or surgeons):** 26.5%  **Age:** not recorded  **Ratio of females to males:** 10 to 22  **Race/ethnicity:** not recorded  **Setting of surgery:** not recorded  **Type of surgery:** hepatobiliary, colorectal, orthopedics  **Academic or community:** not recorded | **Purpose:** Explored how and why clinicians defer and avoid emergency care and treatment planning (ECTP) conversations.  **Design:** ethnographic study  **Single or multi-centre:** multi | ECTP conversations should attend to patients’ and relatives’ emotions. Physicians should commit sufficient time for an in-depth discussion. Time constraints led surgeons and geriatricians to defer these conversations. Emergency and acute medicine clinicians deferred conversations due to the high-turnover ward environment and patients’ acute conditions, leading clinicians to prioritize conversations with those most in need (most acutely sick). |
| 11. Grus & McMullen, 2019 [28] | **Country of publication:** USA  **Number and type of participants:** 8 physicians, 12 patients  **Proportion of participants that were surgical (surgical patients, trainees or surgeons):** 75% of physicians were surgeons, 100% of patients were surgical patients  **Age:** mean of 59.3 (for patients)  **Ratio of females to males:** 12 to 0 (for patients)  **Race/ethnicity:** 91.7% Non-Hispanic White, 8.3% Black (for patients)  **Setting of surgery:** elective or scheduled oncology  **Type of surgery:** breast  **Academic or community:** academic | **Purpose:** Assessed how clinicians balanced sharing medical information and considering patients’ goals and values during breast cancer surgery consultation.  **Design:** Qualitative study. Ethnographic observations of surgeons during discussions and 1:1 interviews with surgeons, radiation oncologists and patients.  **Single or multi-centre:** single | Clinicians prioritized sharing medical information and biomedical facts. Four patient factors were necessary to integrate patient values and goals into conversation: ability to process large quantities of info quickly, willingness to embrace swift decision making, ability to quickly formulate one’s values, and prioritization of surgical choice as the goal of the conversation. |
| 12. Hadler et al., 2016 [29] | **Country of publication:** USA  **Number and type of participants:** 69 physicians  **Proportion of participants that were surgical (surgical patients, trainees or surgeons):** 24.5%  **Age:** not recorded  **Ratio of females to males:** not recorded  **Race/ethnicity:** not recorded  **Setting of surgery:** emergency benign or oncology  **Type of surgery:** vascular  **Academic or community:** academic | **Purpose:** Assessed the attitudes of attending and resident surgeons and anesthesiologists regarding their understanding of and practices for consenting critically ill patients with advance directives in the operating room.  **Design:** survey  **Single or multi-centre:** single | Most critically ill patients “sometimes or always” came to the operating room without sufficient discussion on how their perioperative care might impact their prognosis and long-term GOC (92.2% of respondents). Trainees were less likely to confirm the presence of an advance directive preoperatively. 60% of staff surgeons would decline to perform a case if the patient’s advance directive limited peri/postoperative care. |
| 13. Hu et al., 2022 [30] | **Country of publication:** USA  **Number and type of participants:** 26 physicians  **Proportion of participants that were surgical (surgical patients, trainees or surgeons):** 100%  **Age:** not recorded  **Ratio of females to males:** 11 to 15  **Race/ethnicity:** 7.7% Hispanic, 88.5% Non-Hispanic, 3.8% Unknown  **Setting of surgery:** elective or scheduled oncology  **Type of surgery:** 80.8% surgical oncology, 11.5% colorectal, 3.8% gynecology, 3.8% urology  **Academic or community:** academic | **Purpose:** Explored factors affecting the adoption of quality standards for goal-concordant care in patients with advanced cancer.  **Design:** Qualitative study. 1:1 interview.  **Single or multi-centre:** multi | Surgeons envisioned conversations as a unidirectional flow of information; patients would develop an understanding of surgeon expectations for the procedure, but surgeons did not explicitly ask patients their goals for surgery, such as prolonging life, return to normal activities or alleviating pain. Key factors influencing adoption of quality standards include organizational culture, lack of time, and multidisciplinary collaboration (surgeons believe these discussions would be better had with another clinician/social worker/med oncology/palliative care). |
| 14. Hutul et al., 2006 [31] | **Country of publication:** USA  **Number and type of participants:** 33 residents, 16 staff  **Proportion of participants that were surgical (surgical patients, trainees or surgeons):** 100%  **Age:** not recorded  **Ratio of females to males:** not recorded  **Race/ethnicity:** not recorded  **Setting of surgery:** not recorded  **Type of surgery:** not recorded  **Academic or community:** academic | **Purpose:** Assessed current methods of teaching and attitudes regarding communication skills in surgical residency.  **Design:** survey  **Single or multi-centre:** single | 40% of residents felt comfortable with conducting a family conference, 36% with discussing do-not-resuscitate status, and 24% with discussing transition to comfort care. 30% of residents reported that they received helpful feedback from their attendings. |
| 15. Isenberg et al., 2018 [32] | **Country of publication:** Canada and USA  **Number and type of participants:** 44 patients, 25 physicians  **Proportion of participants that were surgical (surgical patients, trainees or surgeons):** 100%  **Age:** not recorded  **Ratio of females to males:** not recorded  **Race/ethnicity:** not recorded  **Setting of surgery:** elective or scheduled oncology  **Type of surgery:** surgical oncology  **Academic or community:** academic | **Purpose:** Implemented human-centred design to develop an advance care planning decision support video for patients and their family members when preparing for major surgery.  **Design:** human-centred design process  **Single or multi-centre:** multi | Surgeons and patients felt that explicitly referring to advance directives might cause patient distress. While some patients and family expressed a desire for graphic and explicit advance care planning messaging, multiple surgeons were opposed to images of feeding tubes and resuscitation in the advance care planning support video. Surgeons and patients felt that patients should be encouraged to ask about the risks of their surgery. Surgeons play a large role as gatekeepers for comprehending the medical gravitas of decisions. |
| 16. Kalbfell et al., 2021 [33] | **Country of publication:** USA  **Number and type of participants:** 213 patients and family members  **Proportion of participants that were surgical (surgical patients, trainees or surgeons):** 100%  **Age:** mean of 72, standard deviation of 7  **Ratio of females to males:** 91 to 122  **Race/ethnicity:** 83.1% White, 5.2% Black, 3.8% Asian/Pacific Islander, 8.5% Other, 5.2% Hispanic  **Setting of surgery:** scheduled or elective oncology  **Type of surgery:** 12.8% cardiac, 11.1% colorectal, 11.5% hepato-pancreatico-biliary, 12.8% gastrointestinal and sarcoma, 6.6% neurosurgery, 4.9% vascular, 17.7% thoracic, 11.5% urology, 4.4% otolaryngology, 6.6% gynecological oncology  **Academic or community:** academic | **Purpose:** Quantified the frequency of preoperative advance care planning discussion with older patients undergoing major surgery, and characterized how patients and their family members considered advance care planning after postoperative complications.  **Design:** Quantitative analysis and 1:1 interview.  **Single or multi-centre:** multi | 13 of 213 preoperative consultations included discussion on advance care planning. Patients and families felt unprepared for serious complications. Patients found it difficult to translate a list of discrete complications into what their life might be like after a serious complication. |
| 17. Keating et al., 2010 [34] | **Country of publication:** USA  **Number and type of participants:** 4074 physicians  **Proportion of participants that were surgical (surgical patients, trainees or surgeons):** 21.6%  **Age:** mean of 50.1, standard deviation of 9.8  **Ratio of females to males:** 746 to 3328  **Race/ethnicity:** 71.4% Non-Hispanic White, 3.2% Hispanic, 20.6% Asian, 2.9% Black, 2% Other  **Setting of surgery:** elective or scheduled oncology  **Type of surgery:** lung cancer, colorectal cancer  **Academic or community:** not recorded | **Purpose:** Described timing of discussions on end-of-life care, characteristics associated with this, and whether physicians who were more comfortable with addressing end-of-life issues were more likely to discuss these topics while patients were still feeling well.  **Design:** survey  **Single or multi-centre:** multi | 65% of physicians would discuss prognosis “now” (when the patient has 4-6 months to live and is asymptomatic). Fewer would discuss do-not-resuscitate status (44%), hospice (26%) or preferred site of death (21%) immediately, with most physicians waiting for patient symptoms or until there are no further treatments to offer. |
| 18. Kubi et al., 2020 [35] | **Country of publication:** USA  **Number and type of participants:** 200 patients  **Proportion of participants that were surgical (surgical patients, trainees or surgeons):** 78%  **Age:** mean of 62.4, standard deviation of 12.7  **Ratio of females to males:** 100 to 100  **Race/ethnicity:** 29% Non-White, 95.5% Non-Hispanic  **Setting of surgery:** elective or scheduled oncology  **Type of surgery:** 50.6% pancreatic, 12.5% hepatobiliary, 13.4% gastrointestinal, 16.3% other  **Academic or community:** academic | **Purpose:** Described patient preferences regarding timing for advance care planning discussions and past patient experiences with advance care planning.  **Design:** survey  **Single or multi-centre:** single | 43.5% of patients preferred their primary care provider initiate advance care planning discussions vs. 7% preferred their surgeon; this was due to trust and familiarity with their primary care provider. 94% preferred to have discussions early before their prognosis worsened and 45% wished they had been exposed to advance care planning before their cancer diagnosis. Patients reported that engaging in advance care planning and end-of-life discussion is important for minimizing burden on their families, gaining a sense of control, and preventing others’ values from guiding their care. Among Black participants, discussions lacked cultural competency. |
| 19. Llewellyn et al., 2018 [36] | **Country of publication:** United Kingdom  **Number and type of participants:** 15 physicians  **Proportion of participants that were surgical (surgical patients, trainees or surgeons):** 27%  **Age:** mean of 53  **Ratio of females to males:** 2 to 2  **Race/ethnicity:** not recorded  **Setting of surgery:** elective or scheduled oncology  **Type of surgery:** neuro-oncology  **Academic or community:** not recorded | **Purpose:** Elicited key social and structural conditions that contribute to avoidance of advance care planning by clinicians in neuro-oncology.  **Design:** Qualitative study. 1:1 interview.  **Single or multi-centre:** single | Factors contributing to avoidance of advance care planning discussions included the notion that it is a time-intensive practice requiring the right window of opportunity, lack of role clarity, and ambiguities in the definition of advance care planning, its purpose and practice. |
| 20. Margolis et al., 2018 [37] | **Country of publication:** USA  **Number and type of participants:** 20 residents  **Proportion of participants that were surgical (surgical patients, trainees or surgeons):** 100%  **Age:** mean of 28  **Ratio of females to males:** 17 to 3  **Race/ethnicity:** not recorded  **Setting of surgery:** not specified  **Type of surgery:** obstetrics and gynecology  **Academic or community:** academic | **Purpose:** Evaluated the feasibility, effectiveness and satisfaction of an educational intervention for teaching obstetrics and gynecology residents about code status discussions.  **Design:** prospective observational study  **Single or multi-centre:** single | Residents initially felt most prepared to discuss treatment options, and less prepared to discuss hospice, end-of-life care and code status. 78% disagreed that it is solely the responsibility of the attending to address code status, and similarly disagreed (94%) that it is the patient’s responsibility to bring up the topic. |
| 21. Misselbrook et al., 2020 [38] | **Country of publication:** United Kingdom  **Number and type of participants:** 30 patients  **Proportion of participants that were surgical (surgical patients, trainees or surgeons):** 100%  **Age:** not recorded  **Ratio of females to males:** not recorded  **Race/ethnicity:** not recorded  **Setting of surgery:** not recorded  **Type of surgery:** orthopedic  **Academic or community:** academic | **Purpose:** Implemented Recommended Summary Plan for Emergency Care and Treatment (ReSPECT) within an orthopaedic department.  **Design:** quality improvement  **Single or multi-centre:** single | 50% of emergency care and treatment discussions were prompted by an acute deterioration. In most cases, the patient no longer had mental capacity to engage in the process. Many deteriorations and conversations regarding escalation of care occurred out of hours, potentially making conversations more challenging and distressing. |
| 22. Morris et al., 2018 [39] | **Country of publication:** USA  **Number and type of participants:** 20 physicians  **Proportion of participants that were surgical (surgical patients, trainees or surgeons):** 100%  **Age:** median of 45, range of 33 to 63  **Ratio of females to males:** 2 to 18  **Race/ethnicity:** 80% White  **Setting of surgery:** not recorded  **Type of surgery:** 30% trauma, 20% vascular, 50% general surgery  **Academic or community:** academic | **Purpose:** Identified factors affecting surgeon decision-making and the barriers to implementing shared decision-making in emergent settings from a surgeon’s perspective.  **Design:** Qualitative study. 1:1 interview.  **Single or multi-centre:** multi | Surgeons worked to achieve consensus with patients and their families, however they highlighted tension between surgeon judgement and patient autonomy, with a significant responsibility to provide knowledge. Surgeons relied on experience or gestalt to weigh a patient’s risks and benefits, and used an “eyeball test” to consider frailty and establish futility. |
| 23. Murthy et al., 2022 [40] | **Country of publication:** USA and Canada  **Number and type of participants:** 23 physicians  **Proportion of participants that were surgical (surgical patients, trainees or surgeons):** 30%  **Age:** not recorded  **Ratio of females to males:** 5 to 17  **Race/ethnicity:** not recorded  **Setting of surgery:** emergency benign or oncology  **Type of surgery:** orthopedic  **Academic or community:** not recorded | **Purpose:** Determined the knowledge base of physicians on hip fracture prognosis, how physicians’ understanding of hip fracture prognosis informs their communication with patients, practical demands physicians encounter when caring for hip fracture patients, and how physicians navigate these demands to incorporate discussions about prognosis and goals of care.  **Design:** Qualitative study. 1:1 interview.  **Single or multi-centre:** multi | Physicians felt that it was important to discuss prognostic outcomes and the recovery process. They perceived challenges when discussing mortality data with new patients in an acute setting. Physicians usually proposed early surgical intervention to optimize patients’ future prognosis. The pressure to perform surgery soon after patients’ presentation and their responsibility to other patients made it challenging to speak extensively with patients. They focused on discussing risks and benefits of the procedure, and revisited information about recovery, rehabilitation and post-discharge disposition after surgery. |
| 24. Nabozny et al., 2017 [41] | **Country of publication:** USA and Canada  **Number and type of participants:** 43 patients  **Proportion of participants that were surgical (surgical patients, trainees or surgeons):** 100%  **Age:** 9.3% were age 50-59, 34.9% were age 69-69, 46.5% were age 70-79, 7% were age 80+  **Ratio of females to males:** 13 to 30  **Race/ethnicity:** 90.7% White, 4.7% Asian, 4.7% Other  **Setting of surgery:** mixed  **Type of surgery:** 41.9% cardiac, 11.6% intracranial, 9.3% aortic aneurysm, 7% thoracic/esophageal, 9.3% gastrointestinal  **Academic or community:** academic | **Purpose:** Characterized how patients buy-in to treatments beyond the operating room and what limits they would place on additional life-supporting treatments.  **Design:** Qualitative study. 1:1 interview.  **Single or multi-centre:** multi | Patients expressed trust in their surgeon to make decisions about additional treatments if a serious complication occurred, yet expressed preference for significant treatment limitations that were not discussed with their surgeon preoperatively. Patients valued the creation of an advance directive preoperatively. Although patients discussed do-not-resuscitate orders, surgeons did not further explore patients’ personal understanding of what it means to live a “decent life” postoperatively. |
| 25. Pham & Garland, 2021 [42] | **Country of publication:** Canada  **Number and type of participants:** 339 patients  **Proportion of participants that were surgical (surgical patients, trainees or surgeons):** not recorded (“medical and surgical ICU patients”; nursing home cohort included 230 patients, ECMO cohort included 109 patients)  **Age:** mean of 72 and standard deviation of 10.6 for nursing home cohort, mean of 51.8 and standard deviation of 15.8 for ECMO cohort  **Ratio of females to males:** 103 to 127 for nursing home cohort, 45 to 64 for ECMO cohort  **Race/ethnicity:** not recorded  **Setting of surgery:** not specified  **Type of surgery:** endocrine, gastrointestinal, musculoskeletal, neoplastic, obstetrics, otolaryngology, renal, respiratory, vascular  **Academic or community:** academic | **Purpose:** Assessed the quality of end-of-life communication with ICU patients at high risk of death.  **Design:** retrospective cohort study  **Single or multi-centre:** multi | The mean scores for end-of-life communication were low (48.5 for nursing home cohort and 49.1 for ECMO cohort). Communication was worse for those with better prognosis, younger age and better neurological function. Those with better prognosis are at higher risk of receiving care that fails to take their preferences and values into account. |
| 26. Raoof et al., 2017 [43] | **Country of publication:** USA  **Number and type of participants:** 48 residents  **Proportion of participants that were surgical (surgical patients, trainees or surgeons):** 100%  **Age:** not recorded  **Ratio of females to males:** not recorded  **Race/ethnicity:** not recorded  **Setting of surgery:** mixed  **Type of surgery:** general surgery  **Academic or community:** academic | **Purpose:** Investigated the feasibility and utility of a Surgical Palliative Care Immersion Training program.  **Design:** prospective cohort study  **Single or multi-centre:** single | 37.5% received prior palliative care training in medical school and/or residency. 67.5% thought the quality of faculty mentoring in palliative care during residency was poor and 42.5% rarely attended or participated in family meetings with faculty. 80% were unclear on the role of palliative care in surgery. 72.5% felt that it was difficult to initiate discussions of palliative care options in surgery patients. The top three topics that residents felt they needed training on were symptom management, discussing code status and discussing prognosis. |
| 27. Redmann et al., 2012 [44] | **Country of publication:** USA  **Number and type of participants:** 912 physicians  **Proportion of participants that were surgical (surgical patients, trainees or surgeons):** 100%  **Age:** not recorded  **Ratio of females to males:** 51 to 861  **Race/ethnicity:** not recorded  **Setting of surgery:** not specified  **Type of surgery:** 35.9% vascular, 30% neurologic, 34.2% cardiothoracic  **Academic or community:** mixed | **Purpose:** Characterized surgeons’ beliefs and practices around using advance directives in decisions to perform high-risk operations. Explored patient-physician communication about preferences for life-supporting therapies.  **Design:** Qualitative study. 1:1 interview and survey.  **Single or multi-centre:** multi | All surgeons reported discussing the potential for unanticipated outcomes and 95% discussed the need for postoperative life-supporting therapy. 81% discussed patient preferences to limit postoperative life-supporting therapy during informed consent. 52% discussed advance directives before surgery. 54% would decline to operate on patients who have an advance directive that would limit postoperative life-supporting therapy. |
| 28. Schuster et al., 2014 [45] | **Country of publication:** USA and Europe (did not specific countries)  **Number and type of participants:** 22 physicians  **Proportion of participants that were surgical (surgical patients, trainees or surgeons):** 32%  **Age:** not recorded  **Ratio of females to males:** not recorded  **Race/ethnicity:** not recorded  **Setting of surgery:** not specified  **Type of surgery:** not recorded  **Academic or community:** not recorded | **Purpose:** Examined the appropriateness of using decision aids to support advance care planning among high-risk surgery populations and the design of such a decision aid.  **Design:** Qualitative study. 1:1 interview.  **Single or multi-centre:** multi | Physicians highlighted the importance of initiating advance care planning preoperatively when mortality and major morbidity are of concern. Surgery was a trigger for initiating advance care planning because it is a defined moment in which patients will face uncertain outcomes. Challenges for initiating advance care planning included lack of time, difficulty finding an optimal setting, reluctance to acknowledge or discuss death within the field of surgery, and power dynamics in physician-patient relationships. |
| 29. Sokas et al., 2021 [46] | **Country of publication:** USA  **Number and type of participants:** 31 patients  **Proportion of participants that were surgical (surgical patients, trainees or surgeons):** 100%  **Age:** mean of 73.4  **Ratio of females to males:** 15 to 16  **Race/ethnicity:** not recorded  **Setting of surgery:** emergency benign or oncology  **Type of surgery:** 58.1% laparotomy, 38.7% laparotomy with bowel resection, 32.3% cholecystectomy  **Academic or community:** mixed | **Purpose:** Explored older patients’ lived experiences making decisions to undergo emergency general surgery and perceptions of perioperative advance care planning.  **Design:** Qualitative study. 1:1 interview.  **Single or multi-centre:** multi | Patients perceived having no choice but to have surgery. Barriers that limited patients’ ability to meaningfully engage in in-depth conversations included severe symptoms, confusion in the context of rapid onset of illness, and viewing the consent process for surgery as affirmation of their preference to prolong life. Patients were fearful and often could not recall meeting the surgeon, and expressed regret or self-blame for not presenting for help sooner. |
| 30. Steffens et al., 2016 [47] | **Country of publication:** USA  **Number and type of participants:** 35 patients and family  **Proportion of participants that were surgical (surgical patients, trainees or surgeons):** 100%  **Age:** not recorded  **Ratio of females to males:** not recorded  **Race/ethnicity:** not recorded  **Setting of surgery:** not specified  **Type of surgery:** not recorded  **Academic or community:** community | **Purpose:** Designed an intervention to improve perioperative decision making for older adults undergoing high-risk surgery by engaging a council of patients and families.  **Design:** Qualitative study involving a Patient and Family Advisory Council  **Single or multi-centre:** multi | Patients and family were unprepared for surgery and events postoperatively. Patients believed surgery had to be done and were surprised that postoperative recovery was difficult and lacked knowledge about the use of advance directives. Patients reported that their surgeon did not present alternatives that were acceptable to them. Although surgeons named risks and described operations, patients struggled to translate this information into their own life context and assumed they would return to normal postoperatively. |
| 31. Sterie et al., 2021 [48] | **Country of publication:** Switzerland  **Number and type of participants:** 43 patient and physician pairs  **Proportion of participants that were surgical (surgical patients, trainees or surgeons):** 39.6%  **Age:** mean of 83.65  **Ratio of females to males:** not recorded  **Race/ethnicity:** not recorded  **Setting of surgery:** emergency benign or oncology  **Type of surgery:** orthopedic trauma  **Academic or community:** not recorded | **Purpose:** Explored the circumstances in which physicians explain resuscitation and the way these explanations are delivered to patients.  **Design:** Qualitative study. 1:1 interview.  **Single or multi-centre:** single | Resuscitation was explained in 53.8% of conversations. These were brief and concerned technical procedures. These conversations did not include information on the medical indication or prognosis of resuscitation processes. |
| 32. Suwanabol et al., 2018 [49] | **Country of publication:** USA  **Number and type of participants:** 131 physicians  **Proportion of participants that were surgical (surgical patients, trainees or surgeons):** 100%  **Age:** not retired and 70 or younger  **Ratio of females to males:** not recorded  **Race/ethnicity:** not recorded  **Setting of surgery:** scheduled or elective oncology  **Type of surgery:** colorectal cancer  **Academic or community:** not recorded | **Purpose:** Described surgeon perspectives on palliative and end-of-life care for patients with Stage 4 colorectal cancer.  **Design:** survey  **Single or multi-centre:** multi | 76.1% reported no formal education in palliative care. 61.8% cited unrealistic expectations among patients and families as a barrier to discussion on palliation. Other barriers included lack of palliative care specialists, culture to treat, difficulty with prognostication, and fear of legal liability for forgoing life-sustaining treatment. |
| 33. Taylor et al., 2018 [50] | **Country of publication:** USA  **Number and type of participants:** 31 patient and physician pairs  **Proportion of participants that were surgical (surgical patients, trainees or surgeons):** 100% of patients were surgical patients, 55% of physicians were surgeons  **Age:** range of 67 to 95 (for patients)  **Ratio of females to males:** 5 to 26 (for patients)  **Race/ethnicity:** not recorded  **Setting of surgery:** mixed  **Type of surgery:** 23.5% cardiothoracic, 23.5% vascular, 52.9% general and acute care surgery  **Academic or community:** academic | **Purpose:** Characterized patterns of communication that are barriers to goal-concordant care.  **Design:** Qualitative study. Recordings of surgeon-patient/surrogate conversations.  **Single or multi-centre:** single | Surgeons focused on the acute surgical problem and need to make a treatment decision, thus failed to discuss and emphasize the life-limiting nature of the patient’s illness. Surgeons asked patients to express preference for a specific treatment without gaining knowledge about the patient’s priorities or exploring how patients might value specific health states or disabilities. Surgeons struggled to integrate patients’ goals and values to make a treatment recommendation and instead, presented options and noted “it’s your decision.” |
| 34. Thomasson et al., 2011 [51] | **Country of publication:** USA  **Number and type of participants:** 133 patients  **Proportion of participants that were surgical (surgical patients, trainees or surgeons):** 100%  **Age:** mean of 62, range of 19 to 102  **Ratio of females to males:** not recorded  **Race/ethnicity:** not recorded  **Setting of surgery:** emergency benign or oncology  **Type of surgery:** general and trauma surgery  **Academic or community:** academic | **Purpose:** Evaluated the quality of the consent process for de-escalation of care in patients on a general and trauma surgery service who died in-hospital.  **Design:** retrospective cohort study  **Single or multi-centre:** single | 11% of patients were able to participate in a discussion regarding their end-of-life care. 23 patients were initially competent and lost their ability to participate in discussions after a debilitating event. Most of the patients who died underwent de-escalation of care. |
| 35. Udelsman et al., 2019 [52] | **Country of publication:** USA  **Number and type of participants:** 33 physicians  **Proportion of participants that were surgical (surgical patients, trainees or surgeons):** 24.2%  **Age:** not recorded  **Ratio of females to males:** 21 to 12  **Race/ethnicity:** not recoded  **Setting of surgery:** mixed  **Type of surgery:** 50% acute care surgery, 25% surgical oncology, 12.5% vascular, 12.5% thoracic  **Academic or community:** academic | **Purpose:** Explored clinician perspectives on the barriers and facilitators for clinician-to-clinician communication and delivery of goal-concordant patient care.  **Design:** Qualitative study. 1:1 interview.  **Single or multi-centre:** single | All clinicians felt responsible for honouring patients’ GOC. Barriers to clinician-to-clinician communication included inaccessible records, lack of protocols and difficulty documenting complex conversations. Clinicians cited the impracticality of going through every conceivable possibility with a patient and noted that it is better to go through treatments which are consistent with an acceptable quality of life. Language and cultural differences were also barriers to GOC discussions. Facilitators included recognizing patient’s unique treatment priorities and family members with a unified understanding of a patient’s GOC. |
| 36. Ward et al., 2021 [53] | **Country of publication:** USA  **Number and type of participants:** 26 residents, 30 staff  **Proportion of participants that were surgical (surgical patients, trainees or surgeons):** 100% of residents were surgical residents. Did not specify the proportion of staff that were surgeons (30 staff surgeons, nurse practitioners and physician assistants).  **Age:** not recorded  **Ratio of females to males:** not recorded  **Race/ethnicity:** not recorded  **Setting of surgery:** mixed  **Type of surgery:** 53.6% general surgery, 14.3% transplant, 12.5% vascular, 7.1% trauma, 5.4% breast, 3.6% colorectal, 3.6% bariatric  **Academic or community:** academic | **Purpose:** Explored deficits in surgical providers’ understanding of the scope of palliative medicine.  **Design:** survey  **Single or multi-centre:** multi | 45% of respondents reported no training in palliative care. As trainees progressed through residency, they had more involvement in discussions regarding end-of-life. Trauma and critical care specialists reported more training in palliative medicine skills than other specialties. |
| 37. Winner et al., 2016 [54] | **Country of publication:** USA  **Number and type of participants:** 205 physicians  **Proportion of participants that were surgical (surgical patients, trainees or surgeons):** 100%  **Age:** not recorded  **Ratio of females to males:** 41 to 164  **Race/ethnicity:** not recorded  **Setting of surgery:** elective or scheduled oncology  **Type of surgery:** 58.5% surgical oncology, 19.5% hepatopancreaticobiliary, 12.7% transplant, 9.3% breast, 2% endocrine, 2.4% colorectal, 2% thoracic  **Academic or community:** 76.6% academic, 23.4% community | **Purpose:** Explored how surgeons discuss the potential for cancer cure prior to operative treatment, and how surgeons perceive patient priorities and treatment goals.  **Design:** survey  **Single or multi-centre:** multi | 37.6% used the word “cure” in their discussions. 31.7% defined cure as 5-year disease free survival, 31.6% as absence of recurrence over the patient’s lifetime and 10.2% as return to baseline population risk for that specific cancer. 56.9% of physicians perceived that to be cured was among the top 2 priorities of patients. |
